# Detection of Allosteric Effects of lncRNA Secondary Structures Altered by SNPs in Human Diseases

**DOI:** 10.3389/fcell.2020.00242

**Published:** 2020-04-08

**Authors:** Xiaoyan Lu, Yu Ding, Yu Bai, Jing Li, Guosi Zhang, Siyu Wang, Wenyan Gao, Liangde Xu, Hong Wang

**Affiliations:** ^1^School of Ophthalmology and Optometry and Eye Hospital, School of Biomedical Engineering, Wenzhou Medical University, Wenzhou, China; ^2^College of Bioinformatics Science and Technology, Harbin Medical University, Harbin, China

**Keywords:** lncRNA secondary structure, linkage-disequilibrium SNPs, structural heterogeneity, transcription factors, human diseases

## Abstract

Recent studies have shown that structuralized long non-coding RNAs (lncRNAs) play important roles in genetic and epigenetic processes. The spatial structures of most lncRNAs can be altered by distinct *in vivo* and *in vitro* cellular environments, as well as by DNA structural variations, such as single-nucleotide polymorphisms (SNPs) and variants (SNVs). In the present study, we extended candidate SNPs that had linkage disequilibria with those significantly associated with lung diseases in genome-wide association studies in order to investigate potential disease mechanisms originating from SNP structural changes of host lncRNAs. Following accurate alignments, we recognized 115 ternary-relationship pairs among 41 SNPs, 10 lncRNA transcripts, and 1 type of lung disease (adenocarcinoma of the lung). Then, we evaluated the structural heterogeneity induced by SNP alleles by developing a local-RNA-structure alignment algorithm and employing randomized strategies to determine the significance of structural variation. We identified four ternary-relationship pairs that were significantly associated with SNP-induced lncRNA allosteric effects. Moreover, these conformational changes disrupted the interactive regions and binding affinities of lncRNA-HCG23 and TF-E2F6, suggesting that these may represent regulatory mechanisms in lung diseases. Taken together, our findings support that SNP-induced changes in lncRNA conformations regulate many biological processes, providing novel insight into the role of the lncRNA “structurome” in human diseases.

## Introduction

With the development of whole-genome sequencing technology, long non-coding RNAs (lncRNAs) have been studied and discovered to play a key role in complex diseases. LncRNAs regulate gene expression at epigenetic, transcriptional, and post-transcriptional levels (Chen et al., 2019). In lung cancers, HOX antisense intergenic RNA (HOTAIR), a well-studied lncRNA, has been shown to correlate with metastasis and poor prognosis (Loewen et al., 2014; Wang et al., 2018). In addition, aside from regulating expression levels of genes, lncRNA structures govern a complex post-transcriptional regulatory program in diseases (Fujimoto et al., 2016). LncRNAs have been shown to form structural domains that function as landing pads for transcription factors (TFs) to participate in transcriptional regulation (Wang et al., 2017). Since lncRNAs are known to play important roles in various diseases, considerable research has focused on elucidating potential relationships between disease phenotypes and lncRNA structural conformations.

Single-nucleotide polymorphisms (SNPs) are the most common type of variants in the human genome. Functional SNPs not only affect gene expression, but they also influence the structures and stabilities of RNAs (Ramírez-Bello and Jiménez-Morales, 2017). By affecting binding affinities, SNPs regulate gene expression in various diseases at the post-transcriptional level and can thus decrease invasion ability of genes (Halvorsen et al., 2010; Pirooz et al., 2018). Moreover, disease-associated linkage-disequilibrium (LD) SNPs have been predicted to alter the ensemble of RNA structures and to further affect RNA-protein binding sites (Martin et al., 2012). Therefore, investigating haplotypes that include specific pairs of SNPs in high LD may contribute to better understanding pathogenic mechanisms in various diseases.

Recently, lncRNAs have been implicated in several diseases. In addition, many disease-associated SNPs modify the secondary structures of lncRNAs, which affect their expressions and functions, thus leading to the development of diseases (Castellanos-Rubio and Ghosh, 2019). Furthermore, risk variants and their LD SNPs decrease binding affinities of TFs and lncRNAs (Hua et al., 2018). Taking together, known disease-associated or their LD SNPs may cause structural rearrangements of molecules and contribute to disease progression.

**FIGURE 1:**
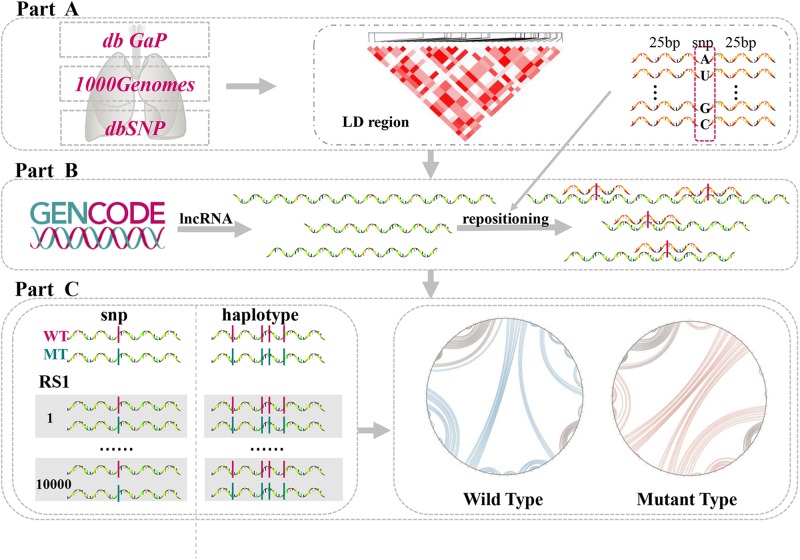
Comprehensive analysis of lncRNA structural heterogeneity generated by linkages-disequilibrium SNPs (LD SNPs). **(A)** Sources of data of lung-disease-associated SNPs and their LD SNPs. **(B)** Information on lncRNA transcripts and positions of LD SNPs within lncRNAs. **(C)** Perturbations to evaluate the differences between WT and MT lncRNAs.

In the present study, we investigated LD SNPs of lung-disease-associated SNPs and mapped them onto lncRNA transcripts across the whole human genome. Connections among single LD SNPs, lncRNAs, and lung diseases were then determined using this methodology. Additionally, the structural heterogeneity of lncRNAs generated by single LD SNPs and their haplotypes were quantified via a computational algorithm. We identified single LD SNPs that significantly altered second structures of lncRNAs. Furthermore, we predicted changes in binding affinities between lncRNAs and TFs. Our comprehensive pipeline was divided into three parts (Figure 1). Collectively, our findings provide further insight into potential molecular mechanisms of lung diseases by demonstrating that lung-disease-associated LD SNPs affect RNA structural rearrangements and concomitantly modulate many biological processes.

## Materials And Methods

### Obtaining and Preprocessing Data

Human disease-associated SNPs were obtained from the Database of Genotypes and Phenotypes (dbGaP), which provided large genetic and phenotypic datasets (Wong et al., 2017). A total of 32 samples of disease phenotypes were downloaded. We identified 42 SNPs associated with lung diseases by searching the following keywords: “lung,” “lung cancer,” and “lung carcinoma.” These SNPs were associated with five types of lung-related diseases, namely, adenocarcinoma of the lung, non-small-cell carcinoma of the lung, small-cell lung carcinoma, lung neoplasms, and squamous-cell carcinoma of the lung. All of these lung-associated genotypes and phenotypes were used for follow-up analyses.

All of the lncRNA-sequence datasets from the whole human genome were downloaded from The GENCODE consortium version 29 (GENCODE V29), which involved comprehensive genomic annotations of lncRNAs that were recruited from GRCh38 (Harrow et al., 2012). Ultimately, 16,042 mature lncRNA genes and 29,566 alternative isoforms were selected for further study.

### Identifying Linkage-Disequilibrium Blocks

LD SNPs can induce substantial changes in the structural ensemble of RNAs (Martin et al., 2012). We identified LD blocks around disease-associated SNPs (LD SNPs), from which we estimated the structural influences of SNPs around lung-disease-associated SNPs. Datasets of SNPs from the 1000 Genomes Project—including chromosome files with genotypes for all of the samples and detailed descriptions of each individual sample—were used as raw LD datasets (Genomes Project et al., 2015; Sudmant et al., 2015). We chose the GRCh38 reference genome to ensure consistency of data sources.

LD blocks associated with lung diseases were extracted as follows. First, samples and SNPs derived from East Asian individuals were selected. Second, only SNPs with two alleles were selected. Third, only SNPs with minor allele frequencies (MAFs) exceeding 5% (common variants) and missing value proportions under 25% were selected; additionally, we required that the SNP genotype of each included sample reach up to 75%. Only samples with *P* values less than 0.01 were selected as significant SNPs. Based on these inclusion criteria and the PLINK toolset, we obtained 42 LD blocks associated with 42 disease-associated SNPs (Purcell et al., 2007).

### Repositioning SNPs in lncRNA Transcripts

Variation analysis of lncRNA transcripts was completed by repositioning SNPs. Bowtie 2, an ultrafast and memory-efficient tool, was applied to map SNPs onto lncRNA transcripts (Langmead and Salzberg, 2012). First, we chose mature lncRNA transcripts as reference sequences. According to the input, Bowtie 2 built a library of long reference sequences. The dbSNP database records sequence information around SNPs (Sherry et al., 2001). The 25-bp upstream and downstream flanking regions of each identified LD SNP were collected from the dbSNP database. Then, at the center of each SNP site, the 25-bp upstream and downstream regions (as short reads) were aligned with lncRNAs. Based on this short-read alignment strategy, we set strict parameters (e.g., end-to-end, –score-min) to ensure precise locations of SNPs. Finally, the output-SAM file contained the symbols of lncRNA transcripts and SNPs, the positions of nucleic acids where matching reads appeared, and the components of the corresponding short reads. We screened start positions both in left and right side of identical lncRNA transcripts. Next, the distance of both ends was used to decide whether SNPs mapped on lncRNA transcripts. The direction of positive and negative in short-read alignment should be taken into account. If the absolute value of distance was 26, it generally indicated SNPs located on lncRNA transcripts.

### Quantifying Structural Heterogeneity of lncRNAs

The exact locations of lung-associated SNPs are a foundation for assessing lncRNA structural disturbances. First, mature lncRNA transcripts downloaded from GENCODE were defined as wild-type (WT) sequences. Meanwhile, lncRNA transcripts with one or more mapped SNPs were assigned as mutant (MT) sequences. Furthermore, we used Linux-based RNA-structure software packages to identify the secondary structures of WT and MT sequences (Reuter and Mathews, 2010). Subsequently, the structural heterogeneity of lncRNAs was quantified via the RNAsmc score designed by our research group, which is the output of an algorithm that computes the difference between two lncRNAs. The stem loop (S), bulge loop (B), interior loop (I), hairpin (H), and multi-branched loop (M) were considered to represent the most essential elements for RNA secondary structures. The locations and amounts of these structural elements were used to calculate the value of the RNAsmc score. The principle of RNAsmc score is as follows:

$$S\,S=\sum_{u\in \left\{S,H,I,B,M\right\}}\frac{u_{p\,1}\,⋂u_{p\,2}}{u_{p\,1}\,⋃u_{p\,2}}+\sum_{u\in \left\{S,H,I,B,M\right\}}\frac{\min\left(u_{n\,1},u_{n\,2}\right)}{\max\left(u_{n\,1},u_{n\,2}\right)}$$

Here, SS is equal to the RNAsmc score which represent the similarity between lncRNA structures; *S*, *H*, *I*, *B*, *M* represents five sub-units as mentioned above; *u*_*p1*_,*u*_*p2*_are the location set of two lncRNA’s base for each kind of sub-units; *u*_*n1*_ and *u*_*n2*_ are the number of each sub-units in each lncRNA structures. We can infer from the scored rules that if there is no difference between two structures, the score is 10; however, if two structures have no overlapping, the score becomes 0. The RNAsmc score was limited to a range of 0 to 10, in which values close to 0 represent a large difference between the two analyzed lncRNA structures, whereas a value of 10 represents structural homogeneity. In addition, in order to show the RNAsmc score was well designed to robustly evaluate the structural heterogeneity, we chose four different score and illustrated their second structure in Supplementary Figure S1. As we expected, the lower score suggested the greater difference between wild-type and mutant lncRNA second structure. This result illustrated that the RNAsmc score was robust.

### Assessing Haplotype-Induced Structural Disturbances of lncRNAs

After assessing the structural heterogeneity of lncRNAs from single SNPs, we next investigated structural transformations induced by haplotype blocks (a series of SNPs within an lncRNA transcript). As we expected, the haplotype was consisted of multiple SNPs in random way. However, the combination among SNPs had not only in reference to linkage disequilibrium, but also closely associated with populations. In population, haplotypes followed special rules to regulate individual biological procedure. Therefore, a comprehensive quality control was essential to acquire haplotypes. First, the annotations of SNPs within lncRNA transcripts from the 1000 Genomes Project were integrated, including the sample, sex, alleles, and genotypes of each SNP. Then, we used PLINK, an open-source toolset for analyzing whole-genome associations, to predict possible combinations of SNPs in the population. In addition, the RNAsmc score was calculated to evaluate structural disturbances by comparing the architectures of WT and MT lncRNA transcripts, which carried haplotype blocks.

### Evaluating Significance of SNP-Modulated Structural Heterogeneity

We further assessed the significance of SNP-modulated lncRNA structural heterogeneity in two ways. First, while keeping the WT and MT SNP sites within lncRNA transcripts unchanged, we performed 10,000 permutations of the flanking sequences of these sites. Additionally, the background distributions of RNAsmc scores between random WT and MT transcripts were calculated and ranked. The *P* value, defined as the Random Score 1 (RS1), was determined by the order of real RNAsmc scores among random scores.

As a second strategy, for a lncRNA sequence with N-bp, we mutated each base into three other bases and obtained all of the possible 3N mutations. The background distributions of scores were computed between the WT sequence and all of the mutated sequences. Subsequently, the *P* value was computed as described above. The mean estimated significance was defined as the Random Score 2 (RS2). In our study, a *P* < 0.05 was used to assign SNPs that significantly altered the conformation of lncRNA transcripts.

### Predicting Variation in Molecular Binding Ability

We evaluated the association between molecular function and modifications in lncRNA conformation. LncRNAs involved in transcriptional regulation of molecular interactions were annotated via manual searching from published papers and LncMAP databases (Li et al., 2018). The LncMAP database has integrated genome-wide transcriptional regulation with paired lncRNAs and gene expressions in pan-cancer. In this database, the regulatory states of lncRNAs and TFs in adenocarcinoma of the lung were detected via transcriptional regulatory network perturbation.

Although the relationships between lncRNAs and TFs are well known, their specific structural interactions are less understood. Here, we used CatRAPID software to predict the interactive region induced by structural units between WT and MT lncRNA transcripts and TFs (Agostini et al., 2013). The intuitive lncRNA secondary structures were visualized by VARNA (Darty et al., 2009). The PDB format of lncRNA transcripts and TFs were obtained by RNAComposer and I-TASSER, respectively (Yang and Zhang, 2015; Biesiada et al., 2016). Additionally, these datasets were then predicted via HDOCK, a web server for protein-RNA docking based on a hybrid strategy (Yan et al., 2017).

## Results

### Mapping SNPs Onto lncRNA Transcripts

**FIGURE 2:**
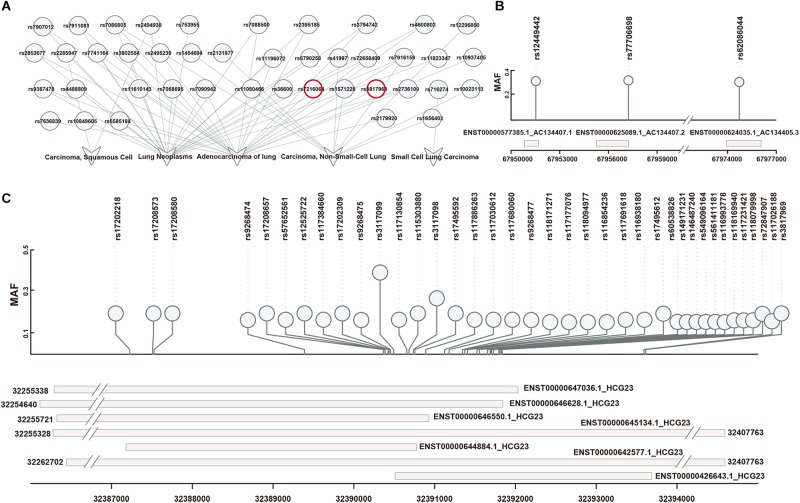
Obtaining and repositioning of LD SNPs. **(A)** Lung-disease-associated SNPs were downloaded in dbGap. SNPs in red represented their linkage disequilibrium SNPs mapped on lncRNAs. **(B)** The one-to-one relationships of LD SNPs and lncRNA transcripts based on a short-sequence alignment algorithm. **(C)** The corresponding locations of LD SNPs mapped onto lncRNA transcripts.

First, 42 SNPs (from an East Asian population) associated with 5 types of lung diseases were downloaded from dbGap (Figure 2A). These SNPs were filtered based on the Hardy-Weinberg Law. Then, we identified LD blocks around disease-associated SNPs (LD SNPs) using PLINK. According to short-read alignments, the LD SNPs were mapped onto lncRNA transcripts in GENCODE V29. We obtained 115 items consisting of 41 LD SNPs (expanded by rs3817963 and rs7216064; red label in Figure 2A), 4 lncRNA symbols (HCG23, AC134407.1, AC134407.2, AC134407.3) with 10 different transcripts, and 1 disease association (adenocarcinoma of the lung; Supplementary Table S1). Three SNPs mapped onto three transcripts, namely, AC134407.1, AC134407.2, and AC134407.3 (Figure 2B). Meanwhile, the lncRNA HCG23, suspected to be correlated with prostate cancer (Eeles et al., 2013), was matched with seven transcripts and 97.39% of all obtained items (Figure 2C). This result suggests that the above four lncRNAs contribute to the onset and development of pan-cancer, or act as necessary regulatory molecules in processes related to adenocarcinoma of the lung. In addition, we found that several SNPs were located in different regions within the same lncRNA transcript, for instance, rs17208657, rs57652561, rs12525722, rs117384660, rs17202309, rs9268475, rs3117099, rs117130854, rs115303880,and rs3117098, all SNPs located in ENST00000646550.1, which may have been due to the distance between each of these linkage SNPs being close to one another. Furthermore, in some cases, one SNP matched with several diverse lncRNA transcripts (Figure 2C). This representation may result from SNPs matched within overlapped fragments of lncRNA transcripts. For example, as shown in Figure 2C and Supplementary Table S1, rs17208657 was mapped onto six lncRNA transcripts (ENST00000642577.1, ENST00000644884.1, ENST 00000645134.1, ENST00000646550.1, ENST00000646628.1, and ENST00000647036.1). These one-to-one correspondences allowed us to explore the effects of LD SNPs on lncRNA transcripts. Additionally, these correspondences suggested that one lncRNA transcript may be influenced by several LD SNPs, or that diverse regulation of different lncRNA transcripts may be generated by identical SNPs.

### Analyzing lncRNA Structural Heterogeneity

**FIGURE 3:**
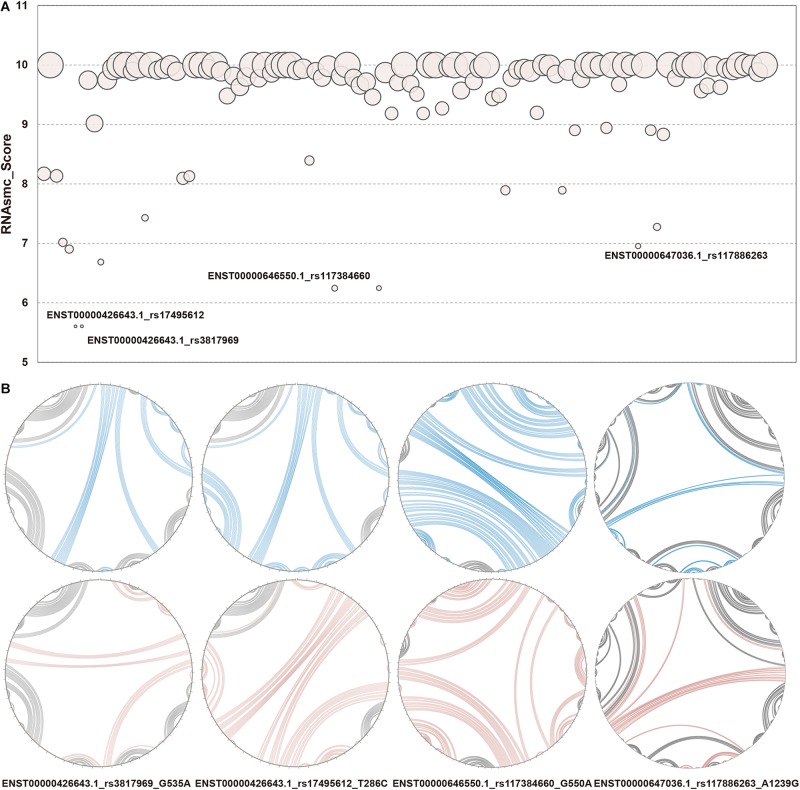
Structural heterogeneity analysis of lncRNA transcripts altered by LD SNPs. **(A)** Quantification of WT and MT lncRNA transcripts. The x axis showed 115 items among LD SNPs, lncRNA transcripts, and lung diseases. The y axis represented RNAsmc scores. The size of each circle indicated the *P* value of the RS1. **(B)** Circular structural comparison of WT and MT lncRNA transcripts. The corresponding relationships between WT and MT lncRNA transcripts and LD SNPs. The lines in blue and red represented the corresponding regions of WT and MT lncRNA transcripts, respectively, altered by LD SNPs. The label under each circle indicated lncRNA transcript, SNP and alleles of SNP. e.g., G535A showed that 535 base G in ENST00000426643.1 change to A.

RNA secondary structure consists of five conformational sub-structures, namely, the stem loop (S), bulge loop (B), interior loop (I), hairpin (H), and multi-branched loop (M). In the present study, we focused on identifying LD SNPs that had an effect on lncRNA secondary structures. We took full advantage of an algorithmic toolkit, RNAsmc score, to probe lncRNA structural heterogeneity based on comparing these sub-structures. We analyzed 115 items that included 41 SNPs in 10 lncRNA transcripts that affected lncRNA secondary structure. The scores of WT and MT lncRNA transcripts were computed and illustrated as bubble charts in Figure 3A, with further information provided in Supplementary Table S2. We found that SNPs of 85 items had an effect on the lncRNA structural ensemble with scores under 10 (about 73.91% of SNPs gave rise to secondary structural variations of lncRNA transcripts), whereas all of the other SNPs (about 26.09%) had no impact, as indicated by their scores of 10. This result suggests that changes in sequences that resulted from SNPs may lead to conformational transformations of lncRNAs. In addition, such disturbances may affect the molecular function of lncRNAs within cells. For instance, changes in lncRNA confirmations may disrupt molecular binding, which may then influence epigenetic, transcriptional, and post-transcriptional regulation of lncRNAs. We found large SNP-induced conformational variations in lncRNAs (Figure 3B), which allowed us to then compare the extent of these SNP-induced structural changes. As shown in Figure 3B, the secondary structures were notably different in WT and MT HCG23 (four different transcripts of HCG23). This result illustrated that the majority of SNPs exhibited an influence on lncRNA secondary structure. Additionally, it is well known that structure often influences function. Therefore, we inferred that LD SNPs not only influence spatial structure, but they also functionally regulate lncRNAs. Furthermore, conformational changes in lncRNA structure may represent a possible cause of lung diseases.

### Comparing and Assessing the Significance of lncRNA Structural Disturbances

**FIGURE 4:**
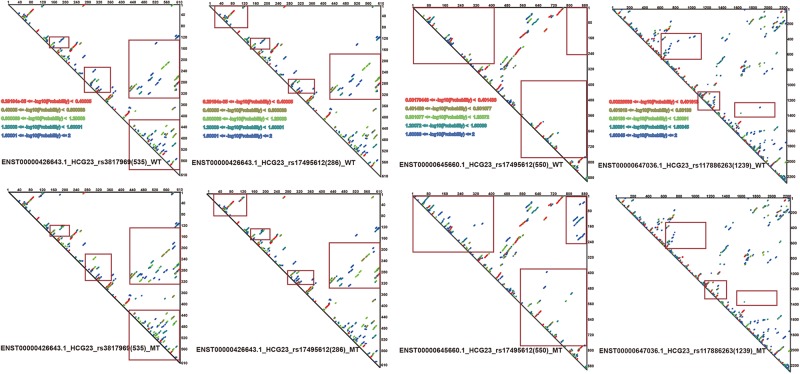
Paired probabilities of WT and MT lncRNA transcripts. Changes in paired probabilities of WT and MT lncRNA transcripts induced by LD SNPs are labeled with red squares.

Since RNAsmc scores alone are not able to determine the significance of lncRNA structural heterogeneity, we next designed two randomized schemes to strictly search for significant SNP-mediated structural changes. The permutation by RS1 and RS2 was illustrated in Supplementary Figures S2A,B. The RS1 was used to calculate *P* values by rearranging flanking sequences of SNPs. In addition, RS2 considered all of the possibilities in the overall length of lncRNA sequences. To evaluate the consistency between RS1 and RS2, we selected items which their RNAsmc score were not 10 (10 means no difference among two structures). Supplementary Figure S2C indicated RS1 and RS2 had identical tendency in evaluating significance of lncRNA structural heterogeneity. And points in red represented significant items appeared by two methods. As determined by both RS1 and RS2, we identified four SNPs that significantly altered the secondary structures of lncRNA transcripts (Figure 3B). Moreover, an additional six SNPs were predicted at a *P* < 0.05 using RS2 (Supplementary Tables S3, S4). In Supplementary Tables S3, S4, although the outputs of significant *P* values between methods were distinct, they exhibited a coherent trend for every item. The RS1 provided an approach to restrict the constitution of each base in lncRNA transcripts; hence, the RS1 was much stricter than the RS2. To ensure reliability of data, we chose common items for evaluation of significance. The base pair probabilities of the four significant WT and MT lncRNA transcripts are shown in Figure 4. These lncRNAs were significantly changed by SNPs, as determined by RS1 and RS2 quantitative analyses. Figure 4 illustrates that a majority of SNPs in lncRNA transcripts only had small effects. Additionally, SNP-induced structural rearrangements often only existed locally (labeled within the red box in Figure 4), rather than affecting overall lncRNA architecture.

### Probing Combined Effects of Multiple SNPs

Comprehensive annotations of SNPs from the 1000 Genomes Project and PLINK toolkit made it possible to predict combined effects of multiple SNPs. Among the 115 items, there were 41 SNPs located in 10 lncRNAs. Meanwhile, only 3 interactions between lncRNAs and SNPs exhibited a one-to-one relationship. This phenomenon suggests that SNP-mediated changes in lncRNA structure are affected by the combined effects of multiple mutation sites. In order to evaluate structural changes induced by multiple SNPs, we mapped SNPs within one lncRNA transcript and predicted possible LD blocks using PLINK. Ultimately, 44 haplotype blocks existed in seven unique lncRNA transcripts. We quantified the overall structural effect of multiple SNPs within one lncRNA transcript by computing the RNAsmc score. The resultant haplotype blocks, RNAsmc scores, and *P* values are presented in Supplementary Table S5. We found that 34 haplotype blocks had an impact on the secondary structures of lncRNA transcripts; however, 10 haplotype blocks had no impact. In addition, we evaluated the significance of lncRNA conformational changes induced by multiple SNPs. Only one haplotype block in HCG23 had a significant effect on lncRNA secondary structure. This haplotype block included 10 SNPs (rs117130854, rs115303880, rs17495612, rs60538826, rs149171231, rs146487240, rs549096164, rs561411181, rs117026188, and rs3817969) mapped onto ENST00000426643.1 (one of the HCG23 transcripts). This result illustrates that a majority of haplotype blocks had only subtle or negligible effects on lncRNA secondary structure. Hence, we inferred that the destructive power of large-span haplotype blocks was very little. In addition, these results demonstrate that the frequency of multiple simultaneous SNP mutations was low.

### Scanning SNP-Mediated Disturbances in Molecular Combined Abilities

We identified four SNPs in HCG23 (including four lncRNA transcripts that significantly affected lncRNA secondary structures and that were associated with adenocarcinoma of the lung). Upon searching published papers and the LncMap database, we identified that five TFs—DDX17, STAT1, PPARG, ETS1, and E2F6—were closely associated with adenocarcinoma of the lung and HCG23. Four of these TFs (DDX17, STAT1, PPARG, and ETS1) have previously been verified to interact with other molecules or to participate in specific signaling pathways (Li et al., 2017; Sun et al., 2017; To et al., 2018; Yang et al., 2019). However, only over-expression of E2F6 has been associated with the development of adenocarcinoma of the lung. Next, we analyzed how perturbations of HCG23 altered binding affinities and structural regulation (Barh et al., 2013). Using catRAPID, the interactive regions between four WT and MT lncRNA transcripts and E2F6 were predicted. Among these predictions, one combination of rs117384660 in ENST00000646550.1 of E2F6 led to noteworthy diversity.

**FIGURE 5:**
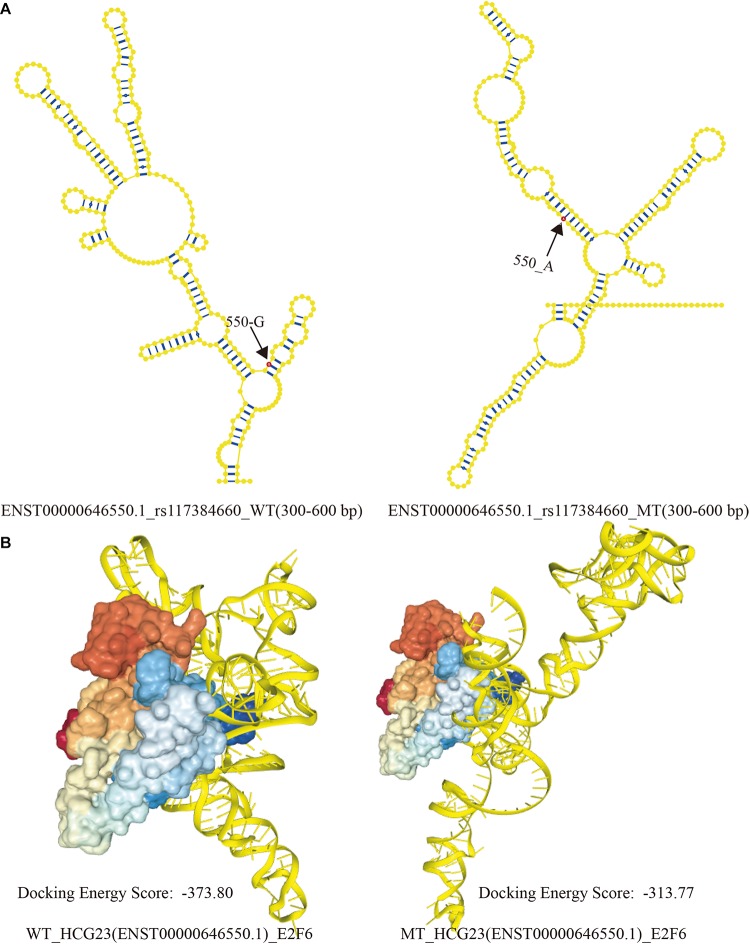
Local structural visualization and prediction of molecular binding. **(A)** The local secondary structures of WT and MT HCG23 (ENST00000646550.1) induced by rs117384660. Bases in red showed the SNP sites in lncRNA transcript, and the numbers indicated location of SNPs. **(B)** Predictions of the structural conformations in interactive regions between WT and MT HCG23 and E2F6 induced by LD SNPs using HDOCK (HDOCK: http://hdock.phys.hust.edu.cn/).

The corresponding intervals of WT and MT lncRNAs were 49–102 bp and 301–352 bp, respectively. However, unique intervals (524–576 bp) arose when base G become A at position 550 of ENST00000646550.1. Based on previous study, Wang et al. proposed that local structural units could be formed within 150–300 bp in a lncRNA transcripts. Then, taking account of binding region predicted by CatRAPID, the interactive region must contain a range of 524–576 bp in lncRNA transcripts. And The SNP of 550 base was exactly located in this region. Therefore, 300 bp (300–600 bp) of the ENST00000646550.1 sequence was chosen to represent the spatial combination with E2F6, as a result of the limitation of RNAComposer. The local secondary structures (300 bp) of WT and MT ENST00000646550.1 are shown in Figure 5A. The visualization of interactive regions was realized by HDOCK. In Figure 5B, we found that the docking score was intuitively distinct. Additionally, when E2F6 was kept at the same angle, the conformations and binding sites varied greatly between WT to MT HCG23. This result suggests that LD SNPs affected the structures of lncRNA transcripts and their abilities to bind to corresponding TFs, which may contribute to the occurrence and development of adenocarcinoma of the lung.

## Discussion

In the present study, we identified LD SNPs by enlarging lung-disease-associated SNPs. We also determined the positions of LD SNPs within lncRNAs, which provided a foundation for establishing the regulatory relationships of LD SNPs and lncRNAs in lung diseases. The LD SNPs in seven different HCG23 transcripts accounted for approximately 97.39% of all analyzed items (Supplementary Table S1). As we known, HCG23 locates at 6p21.32, the HLA locus that is known to be highly enriched for nucleotide polymorphism. Therefore, we developed a strict evaluation system, and set threshold to quantify HCG23 structural heterogeneity induced by single nucleotide mutations. The significance of structural heterogeneity was estimated by RS1 and RS2. RS1 performed 10,000 permutations of the flanking sequences. The *P* value, defined as the Random Score 1 (RS1), was determined by the order of real RNAsmc scores among random scores. However, RS2 mutated each base into three other bases and obtained all of the possible 3N mutations. The significance of scores was computed between the WT sequence and all of the background sequences. Ultimately, only a little SNPs result in significant changes in the structure of lncRNA transcripts. Meanwhile, they might have influence on expression or other functions. These results revealed that HCG23 on chromosome 6 plays a major role in adenocarcinoma of the lung. And according to previous study, HCG23 was also supported participating immune-related diseases (Debiec et al., 2018).

Our analysis of the effects of lung-disease-associated human genetic variation in lncRNAs revealed the extent to which specific SNPs affected lncRNA structure. The RNAsmc score is an algorithm that takes into account the secondary structure of each WT and MT lncRNA. In our present study, 73.91% of SNPs altered the lncRNA structural ensemble. However, we found that a majority of these SNPs exhibited only small or negligible effects on lncRNA structure (Halvorsen et al., 2010; Wan et al., 2014; Zhou et al., 2018). In contrast, only four SNPs had a significant effect on three lncRNA transcripts. These present results are consistent with those of previous studies. In addition, we analyzed the expression of HCG23 which included four significant SNPs. The expression profile in lung adenocarcinoma was derived from The Atlas of ncRNA in Cancer Database (TANRIC) (Li et al., 2015). Using R package-limma, the expression of HCG23 existed significant difference (*P* < 0.05) between normal and lung adenocarcinoma patient. This result can also demonstrated that SNPs affected not only lncRNA second structure, but also gene expression level.

The impact of allelic variants can be determined by analyzing the position and LD block of the associated SNP within an lncRNA sequence; SNPs not only affect gene expression, but they also influence secondary structure (Castellanos-Rubio and Ghosh, 2019). In addition, a previous study demonstrated that a single SNP could alter RNA conformation (Sharma et al., 2019). A similar behavior has been observed for haplotype blocks, the majority of which influence secondary structures of lncRNA transcripts. However, only one analyzed haplotype block significantly affected lncRNA transcripts in our present study. Our results also suggested that LD blocks were not formed by assigning alleles of SNPs randomly, and groups of these LD blocks obeyed specific rules to ensure molecular stability. Hence, we speculate that such conservative metabolic mechanisms for maintaining molecular structure/function may confer self-protection for each individual.

To ascertain whether structural changes affect protein binding, we predicted interactive regions of WT and MT HCG23 with E2F6 using CatRAPID. Compared with that of WT HCG23, MT HCG23 had a distinctive region (524–576 bp). Additionally, we found that binding sites of lncRNAs and proteins changed dramatically (Figure 5). This finding suggests that few LD SNPs inducing structural variation affect protein binding with lncRNAs. Furthermore, structural rearrangement of lncRNAs may contribute to regulation of transcription and/or post-transcription, and contribute to lung diseases.

Structural rearrangements of RNAs play crucial roles in adenocarcinoma of the lung. Rs114020893 in NEXN-AS1 has been predicted to change secondary structure and may contribute to lung cancer susceptibility (Yuan et al., 2016). Additionally, a novel ROS1-ADGRG6 rearrangement induced by the fusion of exons 1–33 of ROS1 on chr6 to exons of 2–26 of ADGRG6 on chr6 has been previously reported in lung cancer (Xu et al., 2019). Therefore, it is important to further elucidate the intricate regulatory mechanisms of disease-associated lncRNAs. Although large numbers of mutations exist within lncRNAs, the mechanisms of such mutations remain unclear. However, the interpretation of non-protein-coding mutations will become more accurate as experimental and computational methods improve.

## Data Availability Statement

The raw data supporting the conclusions in this study will be made available by the authors upon reasonable requests.

## Author Contributions

LX and HW designed the overall concept of the study. XL processed data and wrote the manuscript. YD constructed the graphs. HW and all of the other authors revised the manuscript. All of the authors read and approved the manuscript.

## Conflict of Interest

The authors declare that the research was conducted in the absence of any commercial or financial relationships that could be construed as a potential conflict of interest.

## Supplementary Material

FIGURE S1 The robust analysis of RNAsmc score. Blue and red lines indicate the differences between WT and MT lncRNA transcripts, respectively.

FIGURE S2 The permutation rules and significance evaluating of RS1, RS2, respectively. **(A)** Permutating flanking sequence in random way, and keeping WT and MT SNP allele unchanged. The sequence in blue shadow represented the changed content in RS1. **(B)** Building the background distribution for RS2. The base in red shadow showed it altered in each iteration. **(C)** The significance of lncRNA structural heterogeneity using RS1 and RS2. Red dot indicated that the changes were significant both in RS1 and RS2.

TABLE S1 LD SNPs mapping on lncRNA transcripts.

TABLE S2 The analysis of lncRNA structural heterogeneity induced by SNPs.

TABLE S3 The significance of lncRNA structural heterogeneity by RS1.

TABLE S4 The significance of lncRNA structural heterogeneity by RS2.

TABLE S5 Quantifying and assessing the structural heterogeneity of lncRNA transcripts indeced by haplotypes.
