# Supplementary figures and images for: Detection of Allosteric Effects of lncRNA Secondary Structures Altered by SNPs in Human Diseases
Source: Front Cell Dev Biol. 2020 Apr 8;8:242. doi: 10.3389/fcell.2020.00242 (PMC7156602; doi:10.3389/fcell.2020.00242)

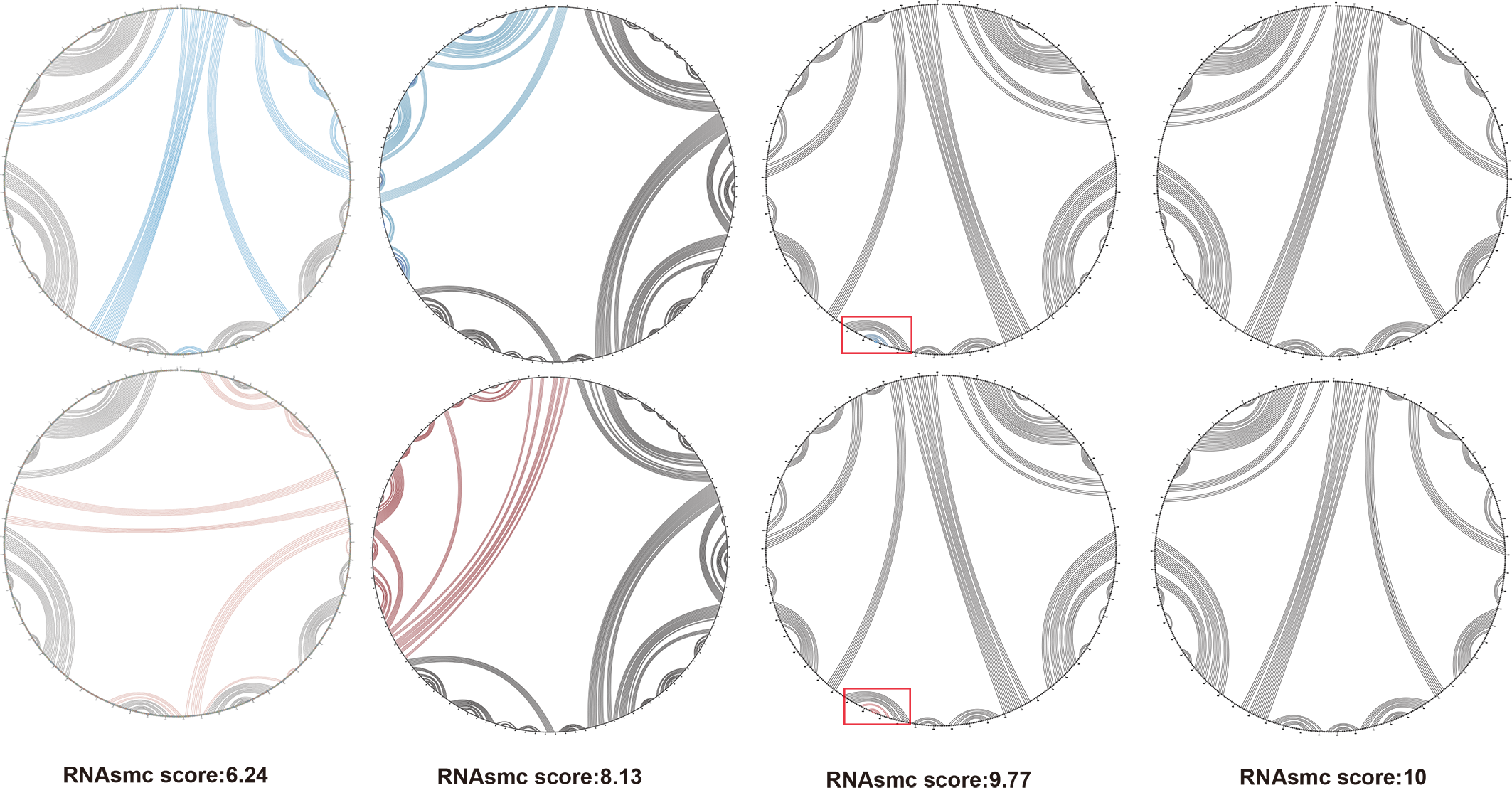

Supplement: FIGURE S1 — The robust analysis of RNAsmc score. Blue and red lines indicate the differences between WT and MT lncRNA transcripts, respectively. [file Image_1.TIF]

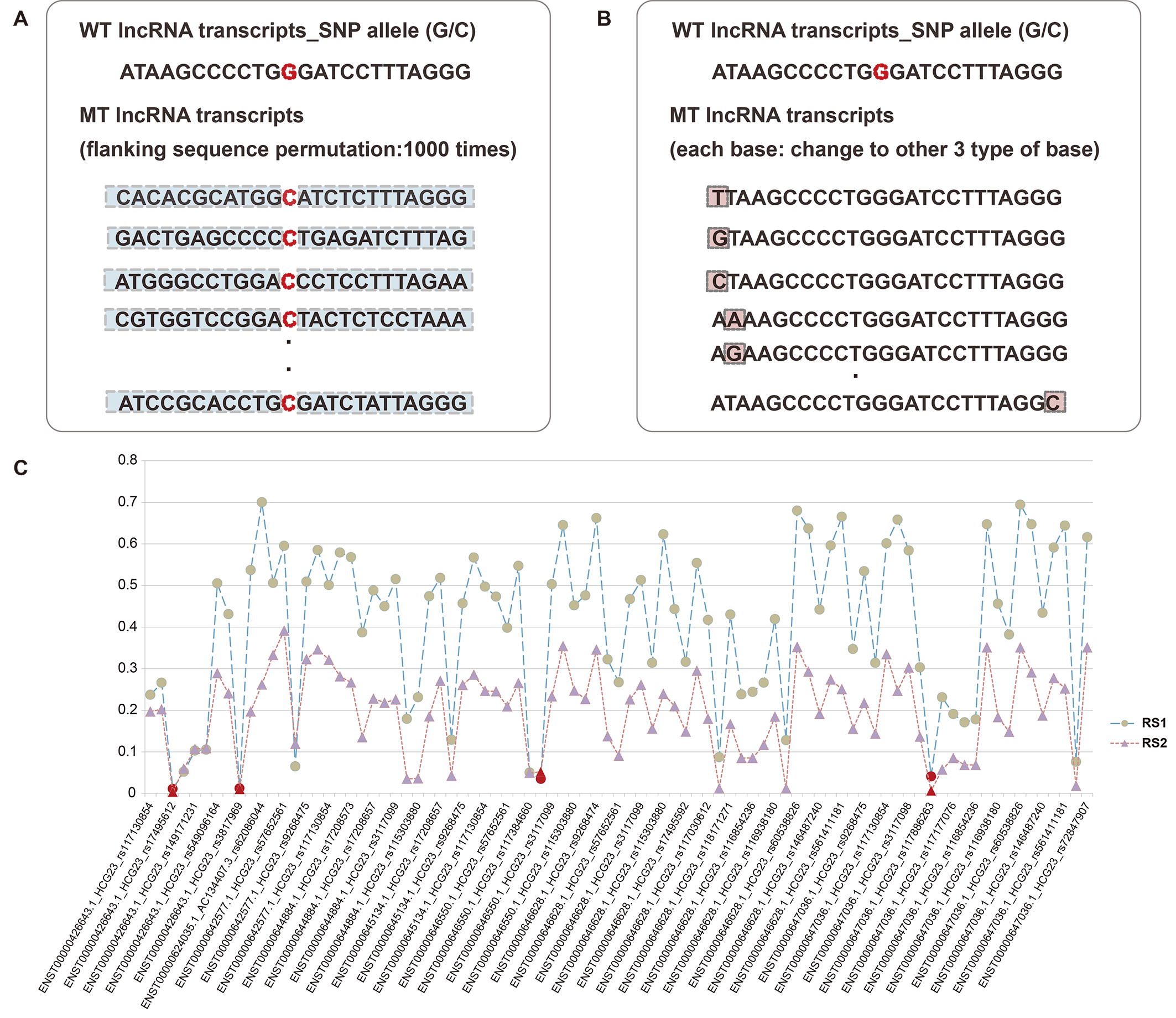

Supplement: FIGURE S2 — The permutation rules and significance evaluating of RS1, RS2, respectively. (A) Permutating flanking sequence in random way, and keeping WT and MT SNP allele unchanged. The sequence in blue shadow represented the changed content in RS1. (B) Building the background distribution for RS2. The base in red shadow showed it altered in each iteration. (C) The significance of lncRNA structural heterogeneity using RS1 and RS2. Red dot indicated that the changes were significant both in RS1 and RS2. [file Image_2.TIF]
